# Effect of Fiber Loading on Thermal Properties of Cellulosic Washingtonia Reinforced HDPE Biocomposites

**DOI:** 10.3390/polym15132910

**Published:** 2023-06-30

**Authors:** Safieddine Bahlouli, Ahmed Belaadi, Azzedine Makhlouf, Hassan Alshahrani, Mohammad K. A. Khan, Mohammed Jawaid

**Affiliations:** 1 Abbes Laghrour University, Khenchela 40000, Algeria; 2 Department of Mechanical Engineering, Faculty of Technology, University 20 Août 1955-Skikda, El-Hadaiek Skikda 21000, Algeria; 3 Department of Mechanical Engineering, College of Engineering, Najran University, Najran 1988, Saudi Arabia; 4 Scientific and Engineering Research Centre, Deanship of Scientific Research, Najran University, Najran 1988, Saudi Arabia; 5 Laboratory of Biocomposite Technology, Institute of Tropical Forestry and Forest Products (INTROP), Universiti Putra Malaysia, Serdang 43400, Selangor, Malaysia; jawaid@upm.edu.my

**Keywords:** Washingtonia fiber, HDPE, thermal stability, DSC/TGA, DMA, TMA

## Abstract

In this research work, we aim to study the effect of the incorporation of vegetable fiber reinforcement on the thermo-mechanical and dynamic properties of a composite formed by a polymeric matrix reinforced with cellulosic fibers with the various Washingtonia fiber (WF) loadings (0%, 10%, 20%, and 30% by wt%) as reinforced material in high-density polyethylene (HDPE) Biocomposites to evaluate the optimum fiber loading of biocomposites. In addition, several characterization techniques (i.e., thermogravimetric analysis (TGA), differential scanning calorimetry (DSC), dynamic mechanical analysis (DMA), and thermal mechanical analysis (TMA)) were used to better understand the characteristics of the new composites prepared. With these techniques, we managed to verify the rigidity and thermal stability of the composites so elaborated, as well as the success of the polymer and the structural homogeneity of the obtained biocomposites. Hence, the biocomposite with the best ratio (HDPE/20WF) showed a loss modulus (E″) of 224 MPa, a storage modulus (E′) of 2079 MPa, and a damping factor (Tanδ) of 0.270 to the glass transition (Tg) of 145 °C. In addition, thermomechanical analysis (TMA) of the biocomposite samples exhibited marginally higher Ts compared to the HDPE matrix. The best results were recorded with biocomposites with 20% WF, which showed better thermal properties. This composite material can be used as insulation in construction materials (buildings, false ceilings, walls, etc.).

## 1. Introduction

Recently, due to the growing concern for environmental protection and sustainable development, the field of bio-based materials has taken a step forward. As experience has shown that reinforcing polymers with synthetic fibers causes serious problems, namely disposal and recycling, researchers have focused on this topic to find solutions that meet the global trends in environmentally friendly materials [1]. To solve this problem, researchers aim to prepare fully or partially biodegradable composites by replacing synthetic fibers (carbon, glass, etc.) used as reinforcement with natural fibers. These last natural fibers are recognized by their abundance, lightness, low energy consumption, biodegradability, strength, and high specific modulus. With such characteristics, vegetable fibers can be a sustainable solution for creating nature-friendly biocomposites. [2].

Due to the fight against the release of carbon into the environment and the increasing demand for clean energy production and sustainable materials with high performance for use in high-tech engineering applications, composites based on a polymer matrix reinforced with plant fibers are attracting increasing interest. These composites offer great potential in various fields (sports, automotive, buildings, aerospace, prostheses, etc.) where the mastery of certain properties such as strength and lightness is really required [3,4,5]. For example, major automobile manufacturers have begun using biocomposite materials reinforced with plant fibers in vehicle interiors, such as roofs, trunk liners, internal engine covers, dashboards and door panels, sun visors, seat backs, and bumpers. As a result of their lower environmental impact, higher quality features, and possible lower production costs, biofiber/filler-reinforced polymer biocomposites have emerged as a prospective alternative to conventional composites [6,7,8].

The selection of a matrix plays a crucial role in mechanical characterization. There are two types of matrices that are most commonly used, namely thermoplastics and thermosets. In-depth research has been conducted on thermoplastic resins, including polyvinyl chloride (PVC), polypropylene (PP), and polyethylene (PE), to evaluate their mechanical and thermal properties [9,10]. Similarly, studies have been conducted on epoxy and polyester [11]. Indeed, plant fibers have been used by many researchers to reinforce polymer matrices, and investigations have been carried out to assess their mechanical and thermal characteristics [12,13,14]. These works revealed that plant fibers have the potential to improve the mechanical properties of polymer biocomposites, such as tensile strength, stiffness, and impact resistance. Moreover, plant fibers exhibit notable thermal behavior, including low thermal conductivity and high specific heat capacity. Therefore, they have established themselves as a promising alternative to synthetic fibers for reinforcing polymer matrices. Furthermore, natural fibers are abundant, renewable, biodegradable, and cost-effective, making them environmentally friendly and sustainable materials for biocomposite manufacturing [2,15].

Furthermore, the introduction of fillers can cause changes of different magnitudes in the molecular and microscopic properties of the polymer matrix. Polypropylene, a basic type of polyolefin, has attractive characteristics, including a lower cost, a wider heat treatment range, resistance to various chemicals, and thermo-oxidative stability. In addition, examining and verifying the qualities and effectiveness of composites made with natural fillers is essential, particularly when these composites experience recurring strains such as damping. DMA is a widely used method to study these characteristics by subjecting composite materials to a sinusoidal force and observing how they react with temperature change, frequency, and time. The polymer modulus is categorized into E′ and E″. When the temperature rises, the E′ of polymers tends to decrease, while the E″ and Tanδ increase until they reach the Tg. The rigidity of the specific polymer composite is recognized as the cause of the decrease in E′, whereas E″ corresponds to the energy dissipation caused by the viscous phase of the polymer [16].

The research work by K. Senthilkumar et al. [15] analyzes the effect of fiber loadings (between 25 and 45 wt%) and different treatments (NaOH and KOH) on the thermomechanical and dynamic properties of biocomposites made of pineapple leaf plant fibers/polyester (PALF/PE). The recorded results demonstrated that composites reinforced with treated fibers had a higher compressive strength and storage modulus than composites filled with virgin fibers. In addition, TMA analysis showed that biocomposite reinforced with 45% fiber exhibited the least dimensional change, signifying better dimensional stability.

The study, led by Saba et al. [17], conducted a comparison between different hybrid nanocomposites (oil palm nanofillers/epoxy and kenaf/epoxy) and analyzed the dynamic mechanical properties. The reinforcement of 1.0 wt.% of oil palm nanofillers improved the E′ and E″ while reducing damping properties. The hybrid nanocomposites also exhibited a higher Tg of 81 °C with a peak height of Tan δ of 0.36. The study suggests that oil palm nanofillers can enhance the dynamic mechanical properties of kenaf/epoxy composites. Asim et al. [18] have studied the influence of fiber loading (40–60 wt.%) and silane treatment (concentration of 2% for 3 h) on the thermal and dynamic properties of PALF and KF composites. Results showed that increasing fiber loading led to an increase in glass transition Tg temperature while thermal stability decreased. In addition, fiber treatment improved thermal stability and reduced flammability. According to the DMA results, the E′ of pure phenolic was found to be the lowest. However, when PALF was added to the phenolic composite at percentages ranging from 40% to 60%, the E′ increased for the composite containing untreated and treated 50% PALF and 50% KF. This increase in E′ can be attributed to the rigid properties of the KF reinforcement. Another recent research work carried out by Asim et al. [19] on the same date palm fiber (DPF) studied the effect of DPF loading ratio on the thermo-mechanical properties of phenolic composites. Results showed that as the fiber loading increased, the mechanical properties increased while the thermal stability decreased. The maximum tensile strength and modulus were achieved with 50% fiber reinforcement. Moreover, the addition of DPF improved the impact strength of the biocomposites. The study highlights the low values of Tanδ for the composites made from DPF. However, an increase in the fiber loading leads to an increase in the Tg of the damping factor. Based on the results, it can be deduced that the composites made with a 50% DPF filler have improved thermomechanical properties due to the good adhesion of the fibers within the polymeric matrix and in the interfacial region. Therefore, this material can be a promising candidate for insulation applications in buildings. In another study, Asim et al. [20] investigated the thermomechanical and dynamic properties of kenaf/PALF/phenolic biocomposites. The composites were produced using hot pressing and varying amounts of kenaf and PALF fibers. Indeed, out of all the hybrid biocomposites, the 15%-PALF/35%-kenaf hybrid biocomposites demonstrated the highest values of E′ at 55 °C and E″ at 101 °C. The minimum Tanδ peak intensity was recorded at 128 °C, while the biocomposite hybrid 35%-PALF/15%-kenaf showed a maximum Tanδ peak intensity at 137 °C. Recent work by Shaikh et al. [21] explored the effects of adding date palm nanofiller to PP on its dynamic and thermo-mechanical properties (DMA and TMA). Dynamic mechanical analysis showed enhanced E′ and Tg temperatures. When compared to PP in its solid state, the biocomposites exhibited a decrease in both E′ and E″ with increasing temperature. At 50 °C, the difference in E′ between the biocomposites and PP was only 9%. Conversely, in the melt state, there was an increase in E′ and E″ across the entire range of angular frequencies. The E′ of the biocomposite containing 1 wt% nanofiller was measured at 0.85 MPa, while the E′ of PP at 0.1 rad/s was 0.732 MPa. According to the authors, developed nano-biocomposites exhibit remarkable dimensional stability in a well-defined temperature and frequency range in the solid state while showing the identical viscoelastic behavior of a mixed polymer liquid in the molten state. Another study conducted by Jawaid et al. Jawaid et al. [22] aim to improve the thermal behavior of epoxy hybrid biocomposites loaded with DPF (short fibers 0.8–1 mm) and bamboo fibers (BF). The authors showed that the performance of the hybrid biocomposite was similar to that of a composite made from BF. Moreover, the analysis of natural fibers derived from different regions of the date palm tree revealed that, when compared to BF, these fibers considerably enhanced the thermal stability and thermo-mechanical properties. The use of hybridized fibers in the epoxy-reinforced biocomposites resulted in improved dynamic mechanical properties, surpassing those of single-fiber composites without hybridization. Gheith et al. [23] investigated the influence of plant fiber loading (40%, 50%, and 60% by weight) in the epoxy matrix on the thermal, flexural, mechanical, and dynamic properties of DPF-reinforced epoxy biocomposites. The results demonstrated that incorporating DPF significantly enhanced the flexural strength (32.6 MPa) and modulus (3.28 GPa) of the biocomposite when loaded with 50% DPF. Moreover, the composite showed improved thermal stability with a degradation temperature, and DMA indicated an increase in E′, E″, and Tg, but the 50% DPF loading showed a net improvement compared to the 40% and 60% DPF charges. Sreenivasan et al. [24] have examined the dynamic mechanical and thermal properties of Sansevieria cylindrica/polyester composites, focusing on the effects of fiber length, loading, and different chemical treatments. The results illustrated that the increase in both factors (fiber length and loading) improved the E′ and Tg of the biocomposite. Chemical treatment further enhanced the dynamic mechanical properties. TGA showed improved thermal stability. Khan et al. [25] examined the impact of cellulose nanofibers and nanoclays (montmorillonite and organoclay) on the mechanical, morphological, thermal stability, and dynamic mechanical properties of kenaf fibers/epoxy nano-biocomposites. The incorporation of CNFs and nanoclays (MMT) resulted in improved tensile strength by 103% and 50%, respectively. CNFs also increased flexural strength by 23.5% and storage modulus by 26%, while nanoclays decreased a damping factor (Tan δ) by 30%. The study demonstrated the potential of CNFs and nanoclays as effective reinforcements for kenaf/epoxy composites.

The purpose of this work is to characterize the thermal properties of previously developed systems (reinforced HDPE not treated with different fiber loads). For that, we used different techniques: the thermogravimetric analysis TGA, to determine in particular the temperature of maximum degradation of our systems; the differential scanning calorimetric analysis DSC, to evaluate the rate of crystallinity and the temperatures of transitions of first order (fusion *T_f_*) and second order (glass transition *T_g_*); the dynamic mechanical analysis DMA, to differentiate the phenomena of relaxation and to study the properties; and finally, the thermomechanical analysis TMA. On the other hand, the damping characteristics that are in high demand in various applications, namely automotive components and the development of light structures, are explored through DMA and free vibration.

## 2. Experimental Procedure

### 2.1. Fiber Extraction and Preparation

The Washingtonia filifera (WF) plant, also known as the desert palm or California palm, is a flowering plant endemic to the southwestern United States and Baja California. This plant can grow up to 15 to 20 m tall and 1 to 4 m wide and has a stout, cylindrical trunk with waxy, fan-shaped leaves. However, it is easily cultivated locally in the region of Skikda (Algeria). Indeed, the natural fibers of WF are easy to remove from the leaf because they are visible to the naked eye. After being removed by hand, the fibers are cleaned. In order to increase the matrix-fiber adhesion and mechanical capabilities, the fibers are properly treated with distilled water (immersed for 20 min at 60 °C), washed with distilled water, and dried in an oven at 65 °C for 20 min to remove the impurities stuck in the fiber surface and generate high-quality fibers. The Washingtonia fibers were dried in an oven at 80 °C for 2 h before being incorporated into the HDPE matrix.

### 2.2. Thermoplastic Matrix

In this work, we used samples of HDPE (high density polyethylene), which is a semi-rigid plastic with a linear structure, supplied by the company SABIC Petrochemicals under the reference PPC10642 (purity = 95%, average density of 0.96, and MFI of 0.4). The molecular weight of the HDPE used is 300,000 g/mol.

### 2.3. Elaboration of HDPE/WF Biocomposites

**Figure 1:**
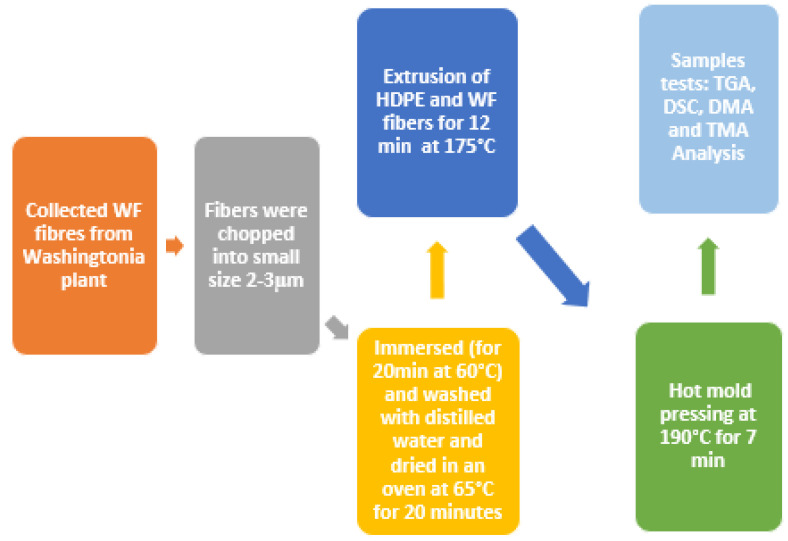
Flowchart of different steps for HDPE-WF biocomposite preparations and tests.

The biocomposite was created using a rolling process with two Thermotron-C. W. Brabender model T303 rouleaux at a temperature of 165 °C for 15 min. To begin, 20% of the weight of HDPE was melted on the rouleaux at 175 °C for 12 min. The WF fibers were then cut between 2 and 5 mm, and the remaining HDPE was added and mixed for 8 min at 65 rpm. The biocomposite was then mixed five to six times for 5 min to improve the homogeneity of the material. Finally, the biocomposite was removed from the rollers and cut to the size of the selected mold. The samples were processed in a mold that was maintained at a temperature of 190 °C for 5 min using a Dake press at a pressure of 10 MPa, and at the end the mold was cooled to 50 °C with cold water (Figure 1). Many formulations developed in this study are cataloged in Table 1.

### 2.4. Thermal Analysis

#### 2.4.1. DSC

The DSC objective is to identify the differential in heat flow established between a sample (E) and a reference (R) while heating or cooling by keeping their temperatures constant. This heat flow is proportional to the heat capacity of the substance under consideration. Whether an endothermic or exothermic event happens during the scan, the flux varies, resulting in a peak on the DSC thermogram. In fact, this approach can be used to study the effect of fiber insertion on the thermal characteristics of the matrix. The heat balance is thus expressed as Q = m Cp ∆T, where Q represents the amount of heat exchanged in joules (J) and m represents the sample’s mass in grams (g). ∆T: difference of the temperature in K; Cp: heat capacity (J/g·K). With an apparatus type Shimadzu DSC-60, we performed thermal studies on the various materials (HDPE and HDPE/WF). A sample amount of 11 to 13 mg was weighed and placed in a sealed aluminum capsule before being placed in the oven. The entire analysis cycle begins with a temperature rise from 25 °C to 200 °C to erase the material’s thermal history, followed by a decline from 200 °C to 25 °C before another rise to 200 °C. The scans (heating and cooling rates) are carried out at 10 °C/min with a nitrogen (*N*_2_) gas flow of 50 mL/min. The endothermic peak was used to determine the crystalline melting temperatures (T_m_) and the crystallinity ratio (Xcr) of the studied samples. A reference value of $∆H_{f-HDPE}^{0}=281\frac{J}{g}$ [26] for the enthalpy of crystallization of 100% crystalline polyethylene was used.

#### 2.4.2. TGA

Thermogravimetry is a thermal analysis commonly used to determine the thermal stability of natural reinforced fiber polymers. In addition, it studies the variation of the mass of a substance as a function of time or temperature. This technique is generally applied to study certain thermal phenomena such as adsorption, desorption, vaporization, decomposition, oxidation, and reduction [27]. TGA analyses were performed by Shimadzu DTG-60H equipment, with the microbalance having an accuracy of ±0.1 μg. Samples of approximately 11 to 13 mg were placed in 70-microliter alumina molds. To characterize the thermal stability of the biocomposites. The samples were heated at 10 °C/min from 25 to 200 °C under a controlled nitrogen atmosphere, and the flow rates were 50 mL/min and 20 mL/min, respectively. Indeed, the peak degradation temperature (T_deg_) represented the temperature at which the rate of degradation was maximal.

#### 2.4.3. DMA

Dynamic mechanical analysis consists of measuring the response of a material following a dynamic loading as a function of frequency and temperature and is also related to the development of the viscoelastic behavior of polymeric and composite materials. It provides useful information on the conservation (E′) and loss (E″) moduli as well as the tangent of the loss angle Tan*δ* (loss factor: Tan*δ* = E″/E′). Indeed, E′ represents the stiffness and the elastic component of the material. The loss modulus E″ represents the viscous component of the material. The viscosity indicates its capacity to dissipate mechanical energy. This behavior is related to the friction of the chains of molecules and their flow, and finally, the loss factor corresponds to the fraction of energy dissipated in viscous form. During our work, we used the NETZSCH DMA-242E ARTEMIS device. The measurement conditions are: 2 °C/min, 25 to 180 °C, and 1 Hz for heating rate, temperature range, and frequency, respectively, with nitrogen as the sweep gas. The HDPE/WF and HDPE samples for DMA in 3-point bending mode have a geometry of 40 × 10 × 2.2 mm^3^.

#### 2.4.4. TMA

The Perkin Elmer TMA-7 apparatus was used to determine the melting temperature (T_m_) and softening temperature (T_s_) of pure HDPE samples and HDPE/WF biocomposites. The samples, which measured 5 mm (length) × 5 mm (width) × 2.2 mm (height), were tested under a nitrogen gas atmosphere (50 mL/min) and an applied force of 5 mN using a penetration probe mode. The tests were conducted in a temperature range of 25 °C to 200 °C with a heating rate of 5 °C/min, and refrigerated cooling was also applied. The results were processed using Pyris v12.1 software.

## 3. Results and Discussion

### 3.1. TGA Analysis

**Figure 2:**
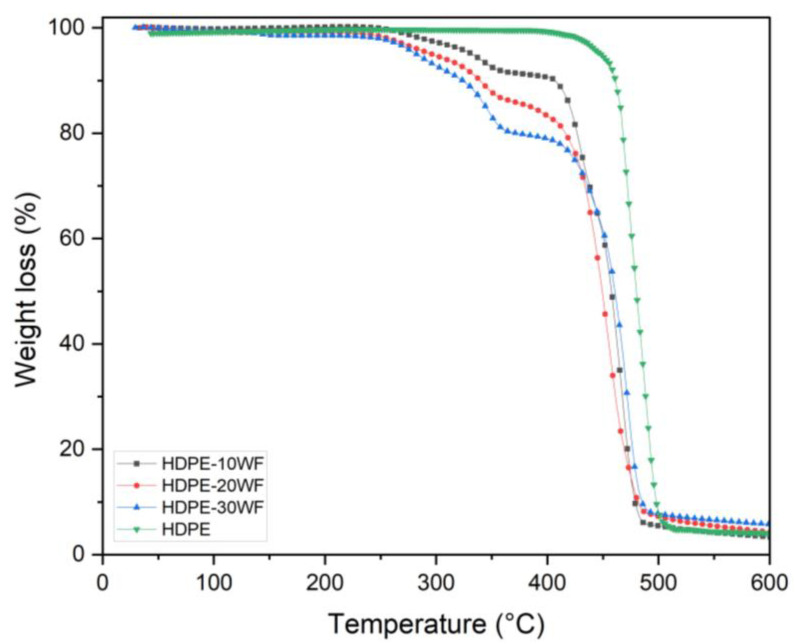
TG curve for different HDPE-WF biocomposites prepared in this work and HDPE.

As described in Section 2.4.2, thermogravimetric analysis allows one to monitor the variation in mass of a sample as a function of temperature and thus access the decomposition parameters of the biocomposite. To facilitate reading, it is convenient to represent the TGA curve of the mass loss in % as a function of temperature in °C. Figure 2 shows the thermogravimetric behavior (TGA) of HDPE and HDPE-WF at different Washingtonia fiber loadings (10, 20, and 30% fiber) for a heating rate of 10 °C/min with an N_2_ atmosphere over a temperature range of 20 °C to 600 °C in order to track their thermal behavior (refer to Table 2). The graph (Figure 2) makes it possible to monitor the thermal behavior of the samples under study and subsequently discuss the effect of incorporating the WF reinforcement into the HDPE matrix on the thermal stability of the resulting biocomposites. The parameters that could account for thermal stability are the initial degradation temperature, the major degradation temperature, the final degradation temperature, and the amount of char formation. Each TGA curve shows two distinct regions of degradation for all biocomposites. The TG curves show that the degradation of HDPE occurs in a single step and starts at 496 °C and ends at a temperature of 518 °C [28]. In contrast, the blend biocomposites reveal that Washingtonia fibers decompose throughout the process, while HDPE begins to decompose at a temperature of 396 °C and experiences the highest rate of weight loss at 480 °C. The TG curve for HDPE indicates that volatile emission begins at 415 °C, and the maximum weight loss rate is recorded at 518 °C. In fact, in all the biocomposites (HDPE-10WF, HDPE-20WF, and HDPE-30WF), the initial degradation occurs within the temperature range of 100–250 °C. This is primarily due to the evaporation of physically weak water molecules on the biocomposites’ surface and dehydration caused by the secondary alcoholic groups [29]. According to the TGA graph, in the case of HDPE-WF composites, the curves demonstrate a two-stage degradation process. The first stage, which takes place between 241 and 378 °C, is attributed to the degradation of WF fibers [28]. Conversely, the primary step involving the degradation of the HDPE matrix occurs in the temperature range of 391–492 °C. The degradation range of lignocellulosic materials typically falls within 150–500 °C. Cellulose exhibits thermal stability within the range of 275 °C to 500 °C, whereas hemicellulose and lignin have degradation ranges of 150–350 °C and 250–500 °C, respectively [30].

Table 2 indicates that weight loss occurs beyond 500 °C, resulting in the breakdown of hydrogen bonds within aromatic polyamides and leading to complete decomposition of the fibers and polymer. The largest amount of residue is shown with the HDPE-30WF and HDPE-20WF biocomposites (7.01% and 5.17%, respectively, at 600 °C). This residue is the result of the thermal degradation of hemicellulose and lignin from cellulose, which resulted in char formation.

### 3.2. DTG Analysis

**Figure 3:**
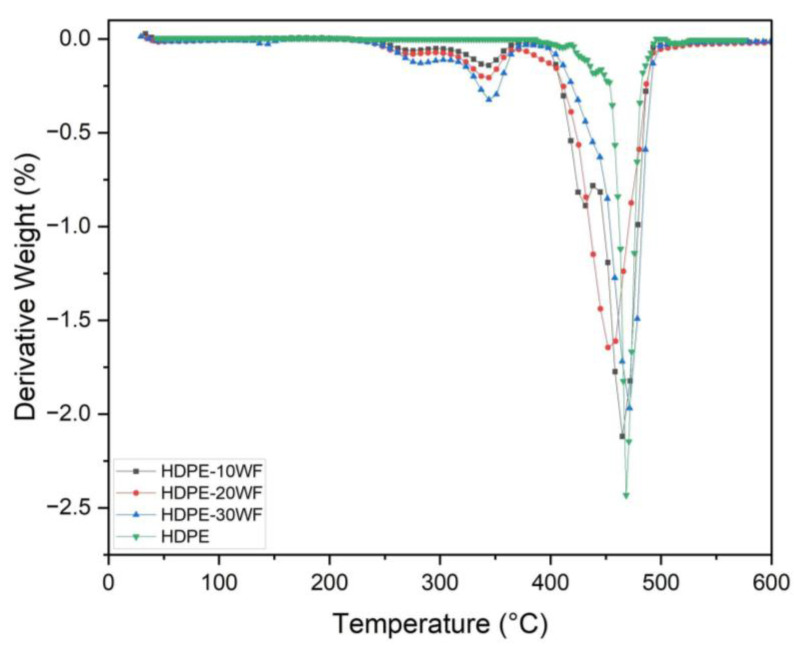
Derivative weight in % as a function of temperature for different HDPE-WF biocomposites and HDPE.

For ease of reading, it is convenient to plot the derived curve DTG of the TGA. This curve makes it easier to identify mass loss phenomena since they are presented as peaks. However, we lose an essential piece of information: the residual mass of the sample at the end of the experiment. Figure 3 shows the behavior of the first derivative of the thermogravimetric TGA curve of HDPE and a comparison of the three biocomposite systems developed in this work. As depicted in Figure 3, better thermal stability of HDPE-WF biocomposites is achieved with a WF fiber reinforcement ratio of about 10% by mass. It should be noted that after this level of reinforcement, the thermal stability of the biocomposite drops, which is why the melting temperature of the HDPE-30WF biocomposite is much lower than that of HDPE (see Table 2).

In the case of biocomposites with 30% reinforcement, the DTG curves do not correspond to what would be obtained by adding the partial contributions of each constituent (Washingtonia fibers and HDPE) since there is a temperature shift. We observe: (i) a low-intensity peak at T = 361 °C, which corresponds to fiber degradation. This shift towards lower temperatures indicates reduced thermal stability of the fibers. This variation is not due to the association with HDPE. It is essential to recall that the fibers in the composite material have already undergone many mechanical and thermal stresses during mixing. (ii) a high-intensity peak at 462 °C, shifted to a higher temperature region than that of the matrix (459 °C), which corresponds to HDPE chain degradation. Consequently, many studies [31] show that beyond 500 °C, polymer chains are completely degraded. In our case, we observe that its decomposition begins around 400 °C and continues until reaching the maximum decomposition temperature of 485 °C. At this temperature, the polymer chains are completely degraded. The decomposition is fully completed beyond 500 °C, and the residual mass represents only 7% of the initial mass in the 30% fiber composite.

### 3.3. DSC Analysis

**Figure 4:**
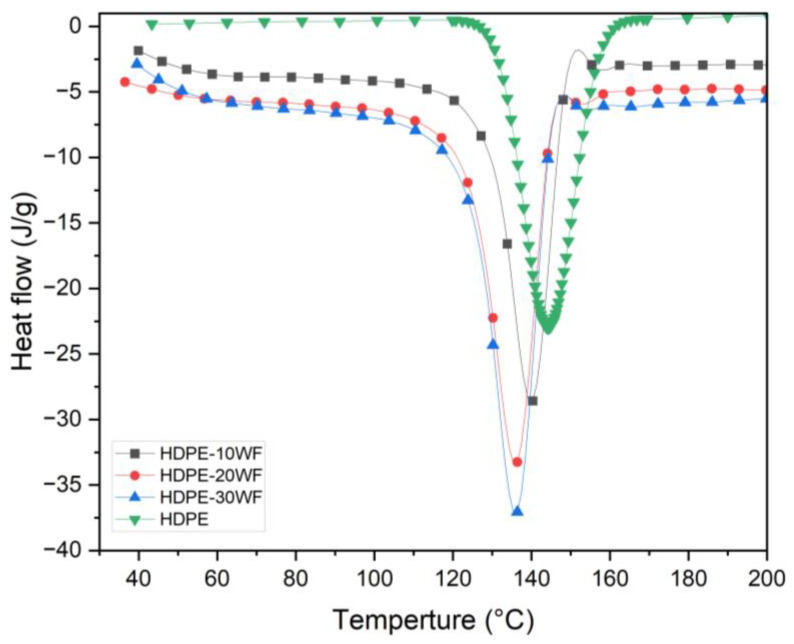
DSC curves for different HDPE-WF biocomposites and HDPE.

Melting is a phenomenon where solid and liquid phases coexist in equilibrium. It corresponds to a constant physical characteristic for each polymer. During melting, crystals absorb energy (an endothermic phenomenon), and this energy corresponds to the enthalpy of fusion (${∆H}_{f}$). The polymer crystallinity ratio $\chi_{r}\left(\%\right)$ is determined by its melting enthalpy. The expression to estimate it is $\chi_{r}\left(\%\right)=100\times \left({∆H}_{m}/\left(1-X\right)\times ∆H\right)$. Or ∆*H*: specific enthalpy of fusion of the perfect crystal (value from literature) and ∆*H_m_* specific enthalpy of fusion of the polymer, determined by integration of the peak of fusion for the polymer. In the case of composites, the mass fraction of the polymer is equal to (1 − X), where X is the fiber ratio. It is therefore necessary to correct this value by the polymer fraction. The DSC analysis was performed according to the conditions detailed in the section dedicated to materials and methods. Figure 4 shows the thermogram of the HDPE and HDPE-WF biocomposites matrix during the temperature rise from 25–200 °C. The curve shape shows that it is a semi-crystalline polymer composed of a single α-crystalline phase. The heat flux evolution shows a single endothermic melting peak (*T_m_* = 144 °C). The same thermogram profile is observed for both raw and processed fiber-reinforced composites. Table 3 presents a summary of the DSC measurements that determined the melting and crystallization behavior of the materials. The results indicate that the crystalline melting temperature of the HDPE matrix in the composites was lower than that of pure HDPE. Furthermore, the *T_m_* values of the composites decreased as the fiber content increased, as indicated in Table 3. This phenomenon could be explained by the strong nucleation that occurred on the fiber surfaces, which reduced the time required for HDPE crystallization. The melting temperatures (*T_m_* = 144, 140, 136, and 136 °C) of all tested samples decreased, and the percent crystallinity of HDPE (%$\chi_{r}$) significantly increased (28.18%, 29.04%, 32.02%, and 39.48%) when reinforced with Washingtonia fibers. This indicates that the fibers have a strong ability to act as heterogeneous nucleating agents [32]. To evaluate the impact of Washingtonia fiber reinforcement on the crystallization behavior and thermodynamics of the HDPE matrix, the thermal crystallization of pure HDPE and its composites was analyzed in the temperature range of 25–200 °C. Furthermore, the addition of WF fiber reinforcement to HDPE improves the thermal stability of HDPE-WF biocomposites, with a saturation ratio of up to 20 percent by mass. Indeed, it is confirmed that HDPE-30WF biocomposite melts at a much lower temperature than HDPE. The biocomposite so generated can be used in a wider variety of contexts, provided that a loading ratio of up to 20% by mass of WF is maintained [33].

### 3.4. DMA Analysis

The DMA test enabled the acquisition of values for parameters such as E′ and Tanδ. Various factors, including the type of resin, reinforcement, and resin interfaces, as well as the interfacial region between them, can impact the viscoelastic properties of composites. Furthermore, the addition of fillers can affect the modulus of polymeric matrix composites. DMA serves as a useful method for analyzing the structural and viscoelastic behavior of materials, with a particular focus on primary relaxations, crosslinking, and density [34]. The E′ obtained from DMA can be correlated with the Young’s modulus and stiffness of the composite material [35]. In addition, the applied energy generates the dissipation of thermal energy, which is related to the E″ [36], and the damping factor, or Tanδ, tells us about the internal friction and provides insight into the internal friction and molecular motion of the materials [36,37]. In this study, DMA was used to evaluate the impact of Washingtonia particles (WP) on the viscoelastic response of HDPE in the solid state.

#### 3.4.1. Storage Modulus E′

**Figure 5:**
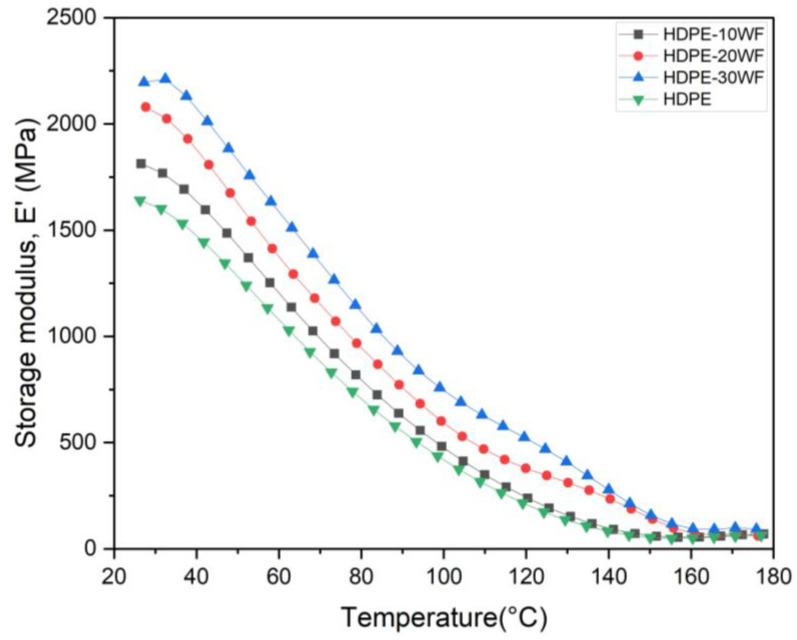
E′ curves for different HDPE-WF biocomposites and HDPE.

The temperature-dependent E′ graph tells us about the stiffness, the interfacial bonding between the fibers and the matrix in the materials, and the degree of cross-linking [36]. E′, which reflects a polymer material’s load-bearing capacity and stiffness, represents the elastic energy accumulated during one oscillation cycle, as previously reported [37,38]. E′ can be divided into three parts: the first is the glassy phase, followed by the transition phase, and lastly the rubbery phase. The first region corresponds to the freezing state, which is very immobile, compact, and tightly packed with the constituents of the composite material [18]. The transition region represents the stage where the storage modulus curve drops after passing through the glass transition temperature (T_g_), as shown in Table 4, due to an increase in polymer chain mobility above *T_g_* [39,40]. Finally, the rubbery region corresponds to a temperature range that causes the material to become more structurally relaxed without any other changes [41]. Storage modulus is generally correlated with Young’s modulus and is largely indicative of a compound’s stiffness, which determines whether a sample is considered stiff or flexible and its tendency or ability to store applied energy for later use [42]. The effect of WP on the storage modulus of HDPE biocomposites (i.e., HDPE, HDPE-10WF, HDPE-20WF, and HDPE-30WF) is presented in Figure 5, and the respective values are listed in Table 4. The results indicate that the E′ of the HDPE biocomposites increased with increasing WP content, with the highest value observed in the biocomposite reinforced with 30% WP. However, the E′ of this biocomposite decreased rapidly with increasing temperature, which can be attributed to the high-density polyethylene matrix’s complex structure and high degree of polymerization. Although initially displaying a lower E′ compared to the composites, the matrix transitioned into the rubbery region as temperature increased, resulting in increased polymer mobility and flow and a reduced reinforcing effect of the fiber on the polymer chain [19]. Asim et al. [18] and Ramakrishnan et al. [43] suggest that a higher E′ value in a sample indicates increased stiffness, as it restricts the movement of matrix chains and enhances bonding between the reinforcement and matrix. The incorporation of particles was found to slightly improve the E′ of HDPE. However, at 150 °C, which is the rubbery region, both HDPE-10WF biocomposites and HDPE showed a lower E′ value. As the temperature increased, the increase in free volume and molecular mobility caused a decrease in E′, with molecules absorbing more energy, resulting in large-scale conformational rearrangements [2]. Although there were no significant differences in E′ observed for HDPE-20WF and HDPE-30WF biocomposites at higher temperatures (rubbery region), E′ was mainly dominated by the intrinsic matrix modulus [44,45]. Previous studies have also shown comparable patterns in the viscoelastic characteristics of natural fiber-reinforced polymers [21,36,46], such as in the cases of PP/date palm nanofiller, PP/yerba mate residues, and treated kenaf-pineapple fibers/phenolic. However, several other aspects, including the structure of the matrix and the nature and amount of fiber present as reinforcement with specific orientations, the appropriate chemical treatment, and the size of the fibers, will likely have a significant effect on the final properties of biocomposites. The temperature-dependent behavior of the biocomposite can be observed in Figure 5, where it is apparent that the E′ values decrease as temperature increases. This decline is due to the biocomposite transitioning from the glassy region to the rubbery region. Within the glassy region, the biocomposites exhibited E′ values in the following order: HDPE-30WF > HDPE-20WF > HDPE-10WF > HDPE. Additionally, Figure 5 reveals that the untreated HDPE-WF biocomposites had the highest E′ peak when the fiber loading was 30 wt%. This result aligns with previous studies [47,48,49], which have also found that E′ peaks at higher fiber loadings. The incorporation of a higher amount of cantante fibers in the polymer resulted in increased stiffness at higher temperatures, as shown by the E′ values of HDPE-WF biocomposites in a temperature range that starts at 50 °C and stops at 100 °C, as per Figure 5. Table 4 indicated a rise in E′ (2211 MPa) at 30 wt% of cantante fibers, owing to improved stress transfer from the fiber to the matrix with a higher fiber loading. However, the value of HDPE-10WF was found to be 1812 MPa. These findings were consistent with the outcomes reported by Indira et al. [50], who used banana fibers as reinforcements for a biocomposite made with a phenol-formaldehyde matrix.

#### 3.4.2. Loss Modulus E″

**Figure 6:**
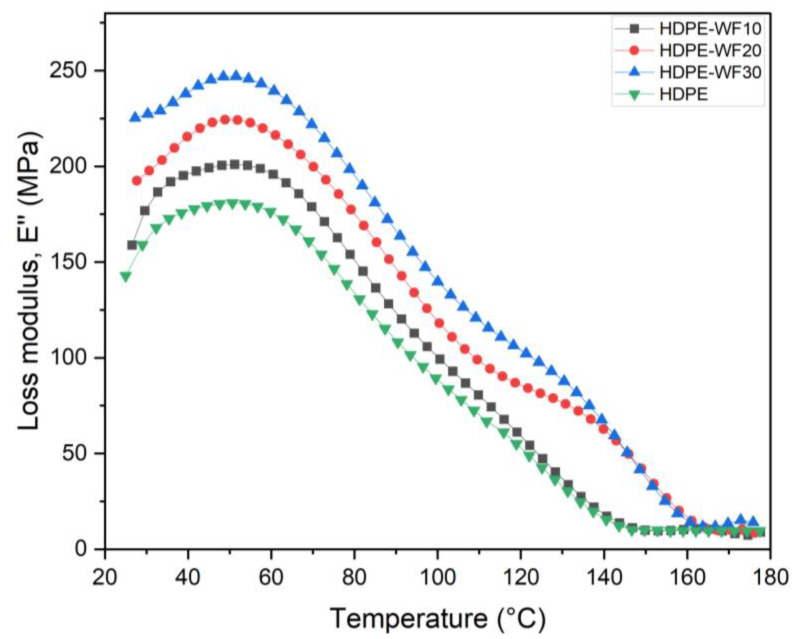
E″ curves for different HDPE-WF biocomposites and HDPE.

The loss modulus E″ is a measure of the energy released by a material in response to sinusoidal loading as a function of temperature [51]. It reflects the damping or viscous properties of the material and indicates the amount of energy dissipated due to molecular mobility. As shown in Figure 6, the E″ of HDPE-WF biocomposites was higher at 30 wt%, indicating increased energy dissipation in the biocomposites due to the addition of fibers. The fiber/matrix interaction in the biocomposites is improved by the addition of fibers, resulting in larger energy dissipation. The E″ values increased in the order HDPE-30WF > HDPE-20WF > HDPE-10WF > HDPE, indicating that the addition of more fibers led to higher energy dissipation. The treatment of the fibers enhances the interfacial bonding between the fiber and matrix, reducing the molecular mobility of the polymeric chains and providing good frictional resistance, leading to higher energy dissipation and greater E″ for composites with treated fibers. This effect has been demonstrated by several researchers, such as in the case of *Sansevieria cylindrica*/polyester [24] and oil palm fibers/linear low-density polyethylene (LLDPE) matrix biocomposites [52].

The graph in Figure 6 demonstrates the temperature-dependent behavior of E″ for HDPE-WF biocomposites and HDPE. The E″ value for HDPE-30WF biocomposites was found to be higher (224 MPa) than that for HDPE-10WF biocomposites, which showed the lowest E″ peak (201 MPa). Furthermore, HDPE-20WF biocomposites showed the highest E″ value in the glass transition region T_g_, followed by HDPE-10WF and HDPE-30WF biocomposites, with values of 51.92 °C, 50.11 °C, and 49.43 °C, respectively. Above 140 °C, the E″ values of HDPE-WF biocomposites appeared to be similar for 20% and 30% loadings, likely due to comparable molecular motions. Similar findings have been reported, indicating that composites with a higher degree of reinforcement tend to exhibit higher *T_g_* values [53]. The low E″ value of HDPE-WF biocomposites with a 20% fiber loading could be due to the fibers stopping the polymer from moving freely, as suggested in previous studies [54]. Moreover, biocomposites containing a weight fraction of 30% WF showed a significantly higher loss modulus, and this is due to the displacement of the WF fiber saturation ratio from the HDPE matrix, which confirms the results obtained by TGA/DSC. Previous research studies [18,19,55] have also reported a similar trend wherein higher weight fractions of fibers (50 wt%) lead to greater energy dissipation due to internal friction and a wider relaxation period. It should be noted that the biocomposites with a 30 wt% loading were the only ones that showed a marginal improvement in loss modulus compared to pure HDPE. A loss modulus analysis can be used to determine the glass transition temperature of biocomposites and HDPE, as shown in Table 4. In this study, the results show that all WP-reinforced biocomposites have a nearly constant glass transition temperature. This observation is in line with the results reported in a previous study [56], which also noted that the inclusion of WP did not significantly affect the glass transition temperature. Furthermore, it is possible that the incorporation of particles within the matrix of a polymer results in a composite with a glass transition temperature almost the same as the starting polymer; this result is observed when the interaction between these particles and the polymer matrix is sufficiently weak [17,57]. In the case where E′ > E″, there is an interconnection of the particles of the natural filler in the HDPE-WF biocomposite [58].

#### 3.4.3. Cole-Cole Plot

**Figure 7:**
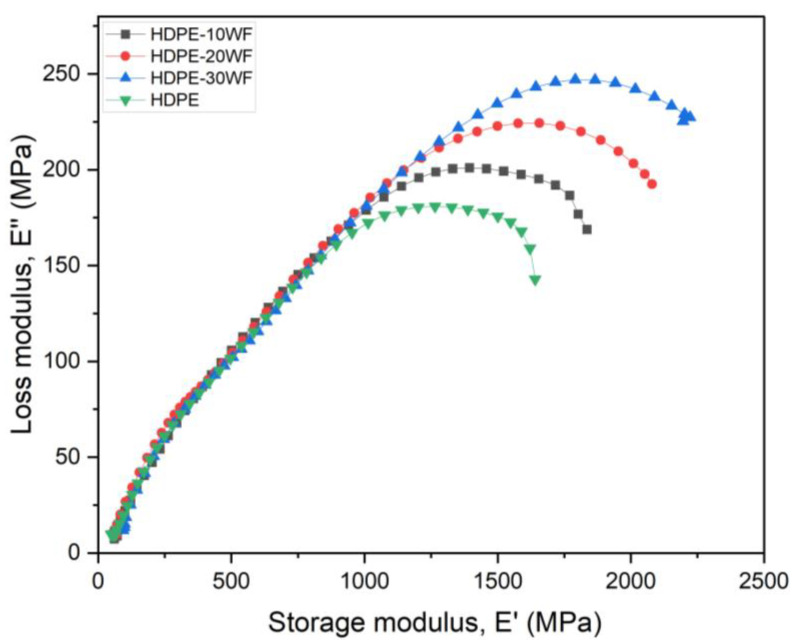
E″ as a function of E′ (Cole-Cole plots) for different HDPE-WF biocomposites and HDPE.

The Cole-Cole plot is a valuable tool for determining the degree of uniformity or non-uniformity in filler dispersion within the polymeric matrix, which represents the homogeneity or heterogeneity of composites. Moreover, it serves as an indicator of poor interfacial bonding between fiber and matrix [59]. Additionally, this curve can be plotted to reveal the relationship between E′ and E″ [17,60,61]. The nature of the Cole-Cole mapping allows for evaluation of the viscoelastic properties and homogeneity of the studied biocomposites [22,62,63,64]. In addition, an irregular shape indicates poor adhesion at the interface caused by heterogeneous phase dispersion in the composite system [65]. It also allows one to determine the structural changes that take place in the cross-linked polymers after their loading by the reinforcement [66]. According to the literature, a smooth, semi-circular, arc-shaped curve is indicative of a homogeneous polymeric system [15,62,64]. Figure 7 shows the Cole-Cole plots of HDPE and HDPE-WF biocomposites, in which E″ is plotted as a function of E′. Observations from the curve indicate that all the biocomposites exhibit a perfect semi-circular shape, which is consistent with a homogeneous polymeric system. Similar behavior was observed as a perfect semi-circle by Joseph et al. [67] in the case of PP/sisal biocomposite. Conversely, various researchers have detected an imperfect semi-circular arc, which indicates heterogeneous filler dispersion in the matrix and good interfacial bonding [68,69]. For instance, basalt-aramid fiber-reinforced phenolic composites [70], ramie-glass fibers in unsaturated polyester [71], and glass/bamboo fiber-reinforced unsaturated polyester resin-based hybrid biocomposites [72] have all displayed such an imperfect arc.

#### 3.4.4. Damping Factor

**Figure 8:**
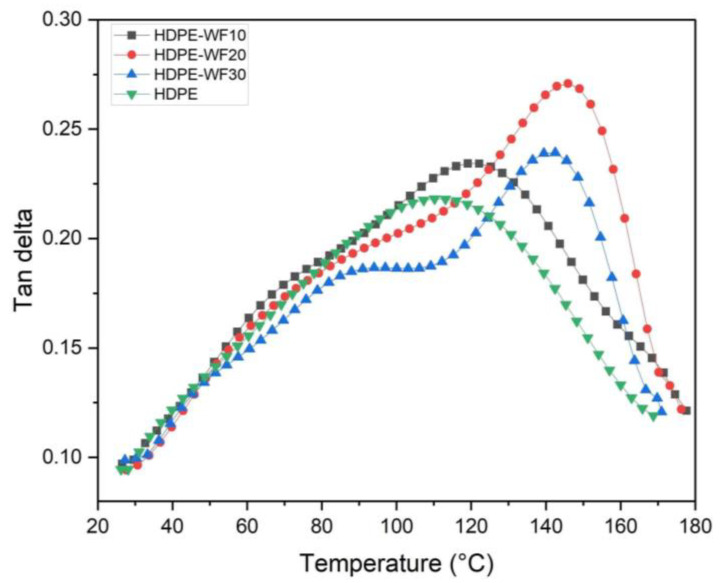
Tan*δ* curves for different HDPE-WF biocomposites and HDPE.

According to the plot in Figure 8, it can be observed that the value of Tanδ increased as the temperature rose, reaching its highest point in the glass transition area before decreasing with a further increase in temperature. The damping factor declined in the rubbery phase of all the biocomposite samples with a rise in temperature, indicating a transition from the frozen stage to the mobility stage of the polymer. At the rubbery stage, the polymer or materials did not have a defined structure remaining [73]. Other studies conducted on cellulosic fibers with polymer biocomposites have reported similar trends [74,75,76].

**Figure 9:**
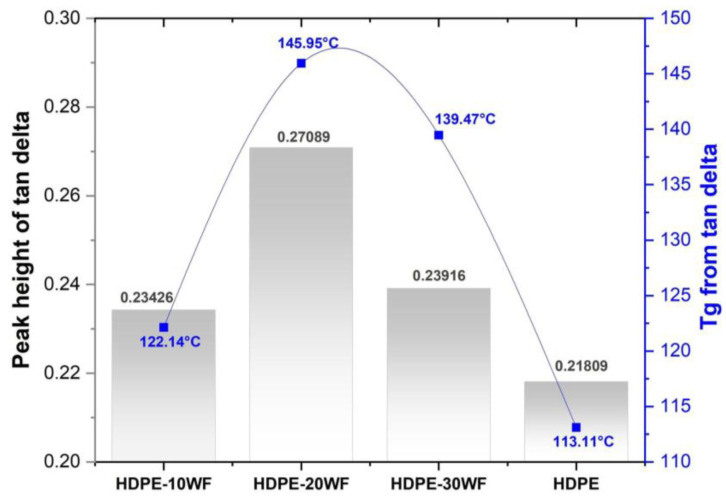
Peak height and T_g_ of HDPE-WF biocomposites and HDPE.

Table 4 and Figure 9 present the peak Tanδ and glass transition temperature values obtained from the Tanδ plots. In the case of HDPE-WF biocomposites, the highest peaks Tanδ values were observed at 20 wt% and 30 wt% loading, with values of 0.27089 (corresponding to a higher T_g_ of 145.95 °C) and 0.23916 (corresponding to a higher T_g_ of 139.95 °C), respectively. Furthermore, the Tanδ values of HDPE-WF biocomposites increased with fiber loading up to 20% and then decreased for the biocomposite loaded with 30% WF fibers (as shown in Table 4). The highest peaks Tanδ and T_g_ values were observed for the HDPE-20WF formulation (at 20 wt% of WF fibers), as shown in Figure 9, indicating poor fiber-matrix adhesion and increased molecular movement in the polymer chains, which result in higher damping characteristics. Conversely, a lower damping factor in the plot indicates improved interfacial adhesions between the reinforcement and polymer, as reported by other studies [62]. The glass transition temperature exhibited an increasing trend in the HDPE-10WF, HDPE-30WF, and HDPE-20WF biocomposites, with values of 145.95 °C, 139.47 °C, and 122.14 °C, respectively. Similarly, the Tanδ values of the composites followed the same pattern. The peak Tanδ value of the biocomposite system varied with the improvement of the reinforcing fibers, which can be explained by the concentration of shear stress in the fibers and the viscoelastic energy loss of the polymeric matrix. This phenomenon has been reported in previous studies [66]. A study of the dynamic mechanical characteristics of several HDPE composites reinforced with natural fibers was made, and it was recorded that a decrease in the values of Tanδ is explained by a more elastic behavior of the composites compared to the HDPE matrix [77]. The reduction in Tanδ values can be attributed to the decrease in viscoelastic lag between stress and strain [78]. The T_g_ obtained from the E″ curve for HDPE-20WF was 51.92 °C, which was relatively lower than the T_g_ obtained from the Tanδ curve (145.98 °C for the same biocomposite, as shown in Table 4). At higher temperatures (100–150 °C), the overlapping of the curves indicated that there was no effect of adding fillers on the viscous dissipation. This trend was also observed in hybrid biocomposites of glass-ramie fibers/polyester [79], glass-pineapple leaf fibers/epoxy [80], and glass-Pennisetum purpureum fibers/epoxy [81]. Generally, the Tanδ allows us to conclude that the glass transition temperature is higher than the loss modulus of 80–100 °C. As the availability of bulk fibers to dissipate vibrational energy is reduced, Tanδ values are higher in biocomposites compared to HDPE.

### 3.5. TMA Analysis

**Figure 10:**
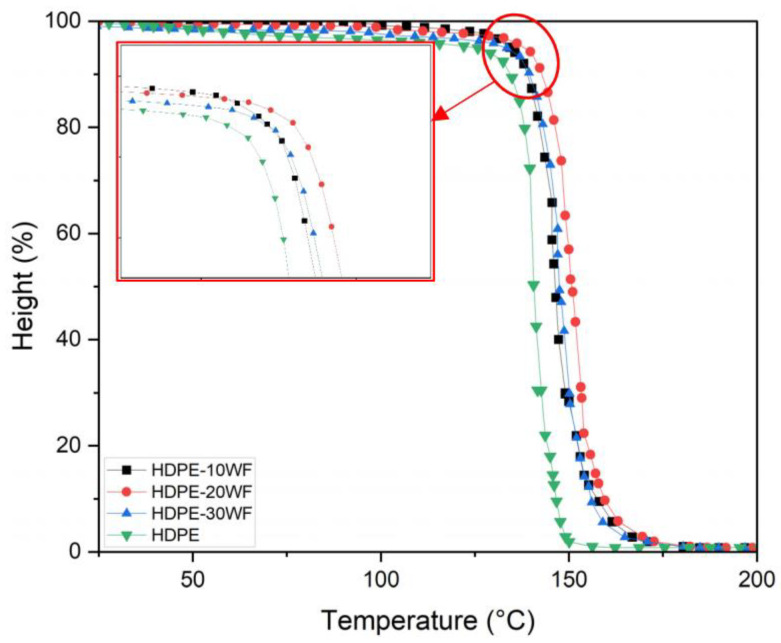
TMA-Height in % as a function of temperature for different HDPE-WF biocomposites and HDPE.

To determine the melt temperature (*T_m_*) or softening temperature (*T_s_*) of both HDPE and HDPE-WF biocomposite samples, changes in probe height (%) were analyzed with respect to temperature (25–200 °C) in the penetration mode using TMA. This method was selected due to its sensitivity and ease of use in measuring both HDPE and HDPE-WF [82] (Figure 10). *T_m_* signifies the initial point at which the sample softens, providing valuable insight into the entanglement between the polymer matrix and the reinforcing materials [83,84]. Typically, the softening temperature increases as the formation of chain entanglements occurs [85]. The *T_m_* of HDPE, HDPE-10WF, HDPE-20WF, and HDPE-30WF biocomposite were 132.16 °C, 137.05 °C, 139.68 °C, and 137.75 °C, respectively. The marginally elevated *T_m_* values observed in the composites may be attributed to the interactions between the fiber and matrix, which can impact the HDPE crystalline structure [86]. As the fiber loading increased, there was a minor rise in *T_m_* for the HDPE-WF biocomposites. However, the *T_m_* values for both HDPE-10WF and HDPE-30WF biocomposites were comparable, indicating that the inclusion of WF particles in the matrix had minimal influence on the *T_m_*. Furthermore, the biocomposite samples exhibited marginally higher *T_s_* compared to the HDPE matrix. This observation may be attributed to the interactions between particles and matrix, which impact the crystalline structure of HDPE. Additionally, the introduction of WP into the biocomposite led to increased elasticity, which likely contributed to the elevated *T_s_* values. Similar findings were observed in the context of HDPE/wood fiber composites [86].

## 4. Potential Applications

The biocomposites made from WF fibers and HDPE possess excellent dynamic mechanical properties, making them a viable replacement for traditional construction materials like aluminum, steel, cement, wood, cladding, and partitioning materials in order to develop sustainable green buildings. Using these materials in construction projects can significantly reduce costs, including material costs, fabrication costs, construction costs, and repair costs.

These biocomposites have a high storage modulus and low damping factor, making them suitable for connecting bridges, stairs, bridge decks, railings, plumbing components, doors, outdoor decking, and door frames. Compared to expensive wooden blocks, they are lighter and have lower damping properties. They are also suitable for low-stress applications such as housing, windmill blades, industrial drive shafts, highway bridge support beams, and paper-making rollers. Furthermore, they have the potential to replace costly materials like stone, concrete, steel, aluminum, and timber in Algeria.

For example, the Building and Construction Division employs HDPE piping systems as ground loops for ground source geothermal applications, which are also referred to as earth energy or geo-exchange systems.

The utilization of HDPE pipes for water systems provides numerous advantages. These pipes are typically manufactured in various diameters and lengths, including straight lengths and coils. HDPE material is resistant to tuberculation and bacterial growth, making it an excellent option for use in harsh environments. Additionally, HDPE pipes exhibit superb chemical resistance properties.

## 5. Conclusions

The addition of Washingtonia fibers with different loadings of 0%, 10%, 20%, and 30% by wt% in a high-density polyethylene matrix resulted in the finding that the addition of WF enhanced TGA, DSC, and DTG thermograms and also improved the thermal stability of HDPE-WF biocomposites compared to HDPE. However, it should be noted that the WF saturation ratio is around 20% by mass, and at higher loads, it is noted that the melting temperature of the biocomposites shifts towards low temperatures (HDPE-30WF) compared to that of HDPE. From the TGA, it can be concluded that the formation of a significant amount of char residues is due to the thermal degradation of hemicellulose and cellulose lignin, and this is noted for the biocomposites HDPE-30WF and HDPE-20WF. According to the DTG analysis, the decomposition begins around 400 °C and continues until reaching the maximum decomposition temperature of 485 °C. At this temperature, the polymer chains are completely degraded. The decomposition is fully completed beyond 500 °C, and the residual mass represents only 7% of the initial mass in the 30 wt% of fiber biocomposite. In addition, the DSC analysis revealed that the melting temperatures (*T_m_* are equal to 144 °C, 140 °C, 136 °C, and 136 °C) for all samples tested decreased, and the percent crystallinity of HDPE and biocomposites (%$\chi_{r}$) significantly increased (28.18%, 29.04%, 32.02%, and 39.48%) when reinforced with Washingtonia fibers. Furthermore, in the DMA analysis conducted in this study, the biocomposite material HDPE-WF, which was reinforced with 30% WP, demonstrated the highest E″ compared to the other biocomposites examined. However, as the temperature increased, the E″ of this biocomposite decreased rapidly. Additionally, an increase in E′ (2211 MPa) was noted in HDPE-30WF, which can be attributed to the enhanced stress transfer from the fiber to the matrix with increased fiber loading. Conversely, the value for HDPE-10WF was found to be 1812 MPa. The Tanδ method determined a T_g_ value that was higher than the loss modulus between 80 °C and 100 °C. The Tanδ values for the biocomposites were higher than those for the HDPE matrix due to a lower volume fraction of fibers available to dissipate the vibrational energy.

## Figures and Tables

**Table 1:** Codification of composition elaborated in this work.

Reference Name	Washingtonia Fibers (wt.%)	HDPE (wt.%)
HDPE	0	100
HDPE-10WF	10	90
HDPE-20WF	20	80
HDPE-30WF	30	70

**Table 2:** TGA results of samples elaborated in this study.

Materials	First Degradation Stage (°C)	Second Degradation Stage (°C)	T (°C) at 10% of Weight Loss	T (°C) at 50% of Weight Loss	Residue (%) at 600 °C
HDPE	396–518	/	459	481	3.98
HDPE-10WF	256–375	398–499	404	460	3.52
HDPE-20WF	241–378	391–492	337	458	5.17
HDPE-30WF	248–389	389–501	323	451	7.01

**Table 3:** DSC results of samples elaborated in this study.

Materials	T_m_ (°C)	T_onset_ (°C)	T_offset_ (°C)	${∆H}_{m}$ (J/g)	***X*_r_ (%)**
HDPE	144	123	165	81.16	28.18
HDPE-10WF	140	106	155	76.33	29.04
HDPE-20WF	136	103	148	92.13	32.02
HDPE-30WF	136	102	148	97.67	39.48

**Table 4:** DMA results obtained for HDPE matrix and HDPE/WF biocomposites.

Materials	Storage Modulus		Loss Modulus		Tan *δ* Peak	
	E′ (MPa)	Tg Value (°C)	E″ (MPa)	Tg Value (°C)	Tan Delta	Tg Value (°C)
HDPE	1642	31.52	180	48.63	0.23	113.11
HDPE-10WF	1812	34.16	201	50.11	0.234	122.14
HDPE-20WF	2079	37.49	224	51.92	0.270	145.95
HDPE-30WF	2211	35.07	246	49.43	0.239	139.47

## Data Availability

Not applicable.
